# Impaired awareness of motor intention in functional neurological disorder: implications for voluntary and functional movement

**DOI:** 10.1017/S0033291717000071

**Published:** 2017-02-10

**Authors:** K. Baek, N. Doñamayor, L. S. Morris, D. Strelchuk, S. Mitchell, Y. Mikheenko, S. Y. Yeoh, W. Phillips, M. Zandi, A. Jenaway, C. Walsh, V. Voon

**Affiliations:** 1 Department of Psychiatry, University of Cambridge, Cambridge, UK; 2 Behavioural and Clinical Neurosciences Institute, Cambridge, UK; 3 School of Clinical Medicine, University of Cambridge, Cambridge, UK; 4 Department of Clinical Neurosciences, Addenbrooke's Hospital, Cambridge, Cambridge, UK; 5 Department of Molecular Neuroscience, UCL Institute of Neurology, London, UK; 6 National Hospital for Neurology and Neurosurgery, UCLH NIHR Biomedical Research Centre, London, UK; 7 Cambridgeshire and Peterborough NHS Foundation Trust, Cambridge, UK; 8 NIHR Biomedical Research Council, Cambridge, Cambridge, UK

**Keywords:** Functional magnetic resonance imaging, functional neurological disorder, inferior parietal cortex, intention, voluntary action

## Abstract

**Background:**

Functional neurological disorders (FNDs), also known as conversion disorder, are unexplained neurological symptoms unrelated to a neurological cause. The disorder is common, yet poorly understood. The symptoms are experienced as involuntary but have similarities to voluntary processes. Here we studied intention awareness in FND.

**Method:**

A total of 26 FND patients and 25 healthy volunteers participated in this functional magnetic resonance study using Libet's clock.

**Results:**

FND is characterized by delayed awareness of the intention to move relative to the movement itself. The reporting of intention was more precise, suggesting that these findings are reliable and unrelated to non-specific attentional deficits. That these findings were more prominent with aberrant positive functional movement symptoms rather than negative symptoms may be relevant to impairments in timing for an inhibitory veto process. Attention towards intention relative to movement was associated with lower right inferior parietal cortex activity in FND, a region early in the processing of intention. During rest, aberrant functional connectivity was observed with the right inferior parietal cortex and other motor intention regions.

**Conclusions:**

The results converge with observations of low inferior parietal activity comparing involuntary with voluntary movement in FND, emphasizing core deficiencies in intention. Heightened precision of this impaired intention is consistent with Bayesian theories of impaired top-down priors that might influence the sense of involuntariness. A primary impairment in voluntary motor intention at an early processing stage might explain clinical observations of slowed effortful voluntary movement, heightened self-directed attention and underlie functional movements. These findings further suggest novel therapeutic targets.

## Introduction

Functional neurological disorder (FND), or conversion disorder, refers to neurological symptoms in the absence of a neurological condition (American Psychiatric Association, 2013). The disorder is common (Carson *et al.* 2000) and equally physically debilitating as Parkinson's disease, with greater impact on mental health and quality of life (Anderson *et al.* 2007; Voon *et al.* 2016). Still, it remains poorly understood. Symptoms are experienced as involuntary but have similar physiological features as voluntary movements (Hallett, 2010). An early crucial study hypothesized that a disturbance of volition might underlie FND (Spence *et al.* 2000). Here we focus on the awareness of motor intention as one aspect of volition, using Libet's clock paradigm (Libet *et al.* 1983).

Libet *et al.* (1983) reported that awareness of the urge or intention to move (W judgement) preceded awareness of the movement itself (M judgement) by around 200 ms. Intention awareness was itself preceded by the *Bereitschaftspotential*, i.e. readiness potential, suggesting an unconscious initiation of the volitional process and constraining the potential for action control (Libet *et al.* 1983; Libet, 1999). This has been widely replicated (Lau *et al.* 2004; Brass & Haggard, 2007; Fried *et al.* 2011). However, Libet emphasized the potential role for action control and the relevance of the interval between W and M judgements in the veto hypothesis: despite unconscious action initiation, action control would be plausible in that interval, allowing for a veto or inhibitory process (Libet, 1999; Haggard & Libet, 2001). Delays in W relative to M judgement have been reported in Parkinson's disease (Tabu *et al.* 2015) and Tourette's syndrome (Moretto *et al.* 2011), suggesting impairments in action control.

The supplementary motor complex (SMC) and inferior parietal cortex (IPC) have been identified as key regions underlying motor intention. Epilepsy patients reported the urge to move when supplementary and pre-supplementary motor areas (SMA, pre-SMA) were electrically stimulated (Fried *et al.* 1991), and single-neuron recordings showed increased firing as the W judgement approached (Fried *et al.* 2011). Attention to intention with Libet's clock also activates the pre-SMA, intraparietal sulcus and dorsolateral prefrontal cortex (dlPFC) (Lau *et al.* 2004). The IPC has been suggested to play a role upstream of the SMC in the development of motor intention with evidence from electrical stimulation, stroke lesion and motor imagery studies (Sirigu *et al.* 2004; Desmurget *et al.* 2009; Desmurget & Sirigu, 2012).

Converging studies implicate impairments in explicit intentional processes in FND. An early study of functional paralysis demonstrated impaired dlPFC activity to attempted movement, which was linked to a disturbance of volition (Spence *et al.* 2000). Functional compared with voluntary tremor has been associated with right temporoparietal junction (TPJ)/IPC hypoactivity, a region implicated in sensorimotor integration (Voon *et al.* 2010). The authors suggested a possible impairment of intentional or prediction processes, as sensory regions were intact. A follow-up study demonstrated SMA hypoactivity in FND subjects during motor preparation, as well as decreased dlPFC–SMA connectivity during internally generated *v*. externally cued action selection (Voon *et al.* 2011). Using Libet's clock, a small study reported that FND patients with functional tremor (*n* = 11) had significantly delayed W relative to M judgements compared with healthy volunteers (HV) (Edwards *et al.* 2011), such that the timing of both judgements was indistinguishable, implying abnormalities in the conscious experience of action underlying functional movements.

In this experiment, we aimed to explore awareness of voluntary motor intention in a larger sample of FND patients with mixed symptoms and HV. We acquired functional magnetic resonance images (fMRI) while participants performed the task devised by Libet *et al.* (1983). We hypothesized that FND patients would have delayed motor intention awareness, and lower IPC and SMC activity compared with HV.

## Method

### Participants

A total of 26 FND patients and 25 HV took part in the study, and one FND patient and one HV were later excluded due to interruption of the experiment during the scan. FND subjects were referred by neurologists and psychiatrists from Addenbrooke's Hospital, Cambridge, and recruited through the FND Hope website (http://fndhope.org/). The diagnosis was based on Diagnostic and Statistical Manual of Mental Disorders, Fifth edition (DSM-5) diagnostic criteria (American Psychiatric Association, 2013) and either made or confirmed by a neurologist from the FND clinic (W.P., M.Z.). Participants were screened by psychiatrists for psychiatric co-morbidities and to document symptom severity (S.M., V.V.). FND subjects with any other major neurological or psychiatric diagnosis, including current major depression greater than moderate severity [Beck Depression Inventory-II (BDI-II) > 17], psychotic or bipolar disorder and substance use disorder were excluded from the study. Current mild depression and elevated depression scores with no current major depression diagnosis were allowed. Recruitment was limited by subjects with head movements, who had ballistic movements, were unable to remain still in the scanner or had claustrophobia. HV were recruited via community advertisements. Participants gave written informed consent and were reimbursed for their time. All experimental procedures were approved by the University of Cambridge Research Ethics Committee.

Participants completed the BDI-II (Beck *et al.* 1961) and Spielberger State-Trait Anxiety Inventory (STAI; Spielberger *et al.* 1983). Pain, motor (paralysis or weakness, non-epileptic seizures, tremor, chorea, tics, gait abnormalities, dystonia, myoclonus) and sensory (somatosensory, vision, hearing) symptoms were recorded based on clinical interview by systematic enquiry, and rating of duration and severity [1 = mild (limited impact on daily functioning); 2 = moderate (noticeable impact on daily functioning with restriction of some activities); 3 = severe (marked impact on daily functioning with restriction of activity in multiple domains); 4 = very severe (impairment in all or virtually all domains of activity)].

### Stimuli and procedure

**Fig. 1.**
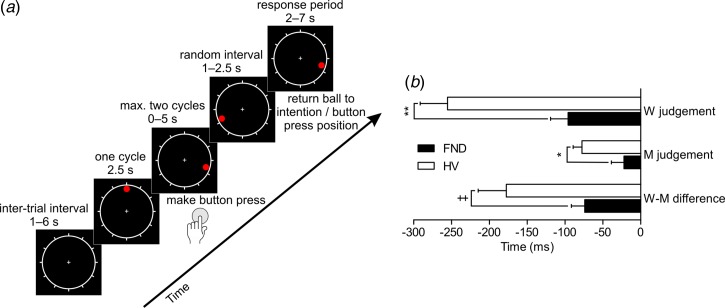
Libet's clock task. (*a*) Schematic representation of the task. The red ball revolved around the unnumbered clock face for a maximum of three cycles; participants had to make a button press after waiting one cycle. The ball continued moving for a random interval, after which participants returned the ball to its position when they had felt the urge (W judgement) or actually pressed the button (M judgement). (*b*) Estimated times of intention (W judgement), movement (M judgement) and difference between intention and movement (W-M) relative to the recorded button press for functional neurological disorder (FND) patients and healthy controls (HV). Values are means, with standard errors represented by the horizontal bars. ^++^ *p* = 0.017, * *p* = 0.009, ** *p* = 0.001. For a colour figure, see the online version of the paper.

In the Libet's clock task (Libet *et al.* 1983; Lau *et al.* 2004) participants watched a red ball revolving around an unnumbered clock face at 2500 ms per cycle (Fig. 1*a*). Participants were instructed to press a button with their left index finger after a random time interval after waiting for one cycle. They were asked to act as spontaneously as possible and in particular to avoid preselecting a position of the ball to trigger the button press. If there was no button press within three cycles the trial was recorded as missed.

The task consisted of two types of trials. In intention trials, participants were asked to attend to when they felt the urge to press the button (W judgement). In movement trials, they were required to attend to when they actually pressed the button (M judgement). After the button press, the ball continued moving for 1000–2500 ms (0.4–1 revolution). Then, it appeared on a random angle on the clock face, and participants were required to return it to the position it was in when they first felt the urge to press the button, or actually pressed it, depending on the type of trial. The final position of the red ball was recorded if still for 2 s or after the 7 s period for responding elapsed. The inter-trial interval lasted 1–6 s. Average latencies of the W and M judgements were calculated as the differences between the final position of the ball and its position at the time of the recorded button press.

Prior to fMRI data acquisition, participants took part in a brief practice session of 10 movement followed by 10 intention trials. The fMRI task consisted of four blocks of 20 trials in the order of movement–intention–movement–intention. The fMRI experiment lasted around 18–21 min.

### MRI data acquisition

MRI data were acquired with a 3 T Tim Trio scanner (Siemens, Germany) with a 32-channel head coil at the Wolfson Brain Imaging Centre, University of Cambridge. Foam pads were used to restrict head movements. Echo planar imaging (EPI) images were acquired in an interleaved fashion [repetition time (TR) = 2.32 s, echo time (TE) = 30 ms, 3 × 3 × 3 mm voxel size, 0.75 mm gap, flip angle (FA) = 78°, 64 × 64 matrix size, 39 slices]. Images were angled 30° to the anterior commissure–posterior commissure line in order to avoid susceptibility signal loss in the orbitofrontal regions. The T1-weighted structural images were acquired using a magnetization-prepared rapid gradient-echo (MPRAGE) sequence [TR = 2.30 s, TE = 2.98 ms, field of view (FOV) = 176 × 240 mm^2^, 1 × 1 × 1 mm^3^ voxel size, inversion time = 1100 ms].

Resting-state fMRI (rsfMRI) data were acquired after the fMRI task for 10 min with open eyes. A multi-echo EPI sequence was used with online reconstruction [TR = 2.47 s, FA = 78°, matrix size 64 × 64, 3.75 mm in-plane resolution, FOV = 240 mm, 32 oblique slices, alternating slice acquisition, slice thickness 3.75 mm with 10% gap, integrated parallel imaging technique (iPAT) factor 3, bandwidth = 1698 Hz/pixel, TE = 12, 28, 44 and 60 ms].

### fMRI data analysis

fMRI data were analysed using SPM8 (http://www.fil.ion.ucl.ac.uk/spm/software/spm8/; Wellcome Trust Centre for Neuroimaging, UK). After slice-timing correction, a mean image for all functional scans was generated for each subject, to which individual volumes were spatially realigned by rigid body transformation. Unwarping was performed during realignment to correct for dynamic motion–distortion interaction artefacts. Structural images were co-registered with the mean EPI image, segmented into grey and white matter, and normalized to the Montreal Neurological Institute (MNI) template. Normalization parameters were then applied to the EPI images for an anatomically informed normalization. Images were then subsampled to 2 × 2 × 2 mm^3^ and spatially smoothed with a 10 mm full width at half maximum (FWHM) Gaussian filter. Scan-by-scan head motions exceeding 0.5 mm in the functional volumes were repaired using Art Repair (Mazaika *et al.* 2007).

The subject-level statistical analyses were performed using the general linear model (GLM). The main events of interest were the 1 s periods prior to the recorded button press in intention and movement trials. Missed trials and the 7 s period for responding were modelled as events of no interest. Vectors containing the event onsets and durations were convolved with the canonical haemodynamic response function, and its temporal and dispersion derivatives. The statistical parameter estimates were computed separately for each voxel for all 12 columns in the design matrix. Movement parameters were also included as regressors of no interest in the GLM in addition to deweighting on the repaired volumes.

Intention *v*. movement trials were contrasted at the individual level and subsequently inputted into an independent-samples *t* test to compare FND patients and HV. Whole-brain voxel-wise group comparisons were performed with a cluster extent threshold of 15 voxels at *p* < 0.001 (uncorrected), correcting for multiple comparisons at *p* < 0.05 assuming an individual-voxel type I error of *p* = 0.01 (Slotnick *et al.* 2003). We also confirmed fMRI activations in *a priori* regions of interest (ROIs) implicated in Lau *et al.* (2004), the pre-SMA, the inferior parietal lobule (IPL) and dlPFC, using small-volume correction as well.

The intention *v*. movement blood oxygen level-dependent (BOLD) contrast was also correlated with the behavioural parameter W–M across all individuals.

### rsfMRI data analysis

rsfMRI data were analysed with multi-echo independent component analysis (ME-ICAv2.5 beta6; http://afni.nimh.nih.gov). First, FastICA was used to decompose multi-echo fMRI data. The BOLD signal is characterized by TE-dependence, which was measured using the pseudo-*F* statistic *κ*; whereas TE-independence was measured by the pseudo-*F* statistic *ρ*. Components were then categorized as BOLD or non-BOLD based on their *κ*- and *ρ*-value weightings, respectively (Kundu *et al.* 2012), and non-BOLD components were removed by projection. Denoised EPI images were co-registered to MPRAGE, normalized to the MNI template, spatially smoothed with a 6 mm FWHM Gaussian filter, and temporally band-pass filtered between 0.008 and 0.09 Hz. Anatomical scans were segmented into grey matter, white matter and cerebrospinal fluid (CSF), and significant principal components in white matter and CSF regions were identified and removed following the CompCor strategy (Behzadi *et al.* 2007).

ROI-driven functional connectivity was computed with the CONN-fMRI Functional Connectivity toolbox (Whitfield-Gabrieli & Nieto-Castañón, 2012) for SPM8. Based on the task-based results, the rsfMRI analysis was seeded within the right IPC, as defined in the Automated Anatomical Labelling atlas.

ROI-to-voxel whole-brain connectivity maps were computed and entered into full factorial GLMs to compare whole-brain connectivity patterns between FND patients and HV. Whole-brain voxel-wise group comparisons were performed with a cluster extent threshold of 15 voxels at *p* < 0.001 (uncorrected), correcting for multiple comparisons at *p* < 0.05 assuming an individual-voxel type I error of *p* = 0.01 (Slotnick *et al.* 2003).

### Ethical standards

All procedures contributing to this work comply with the ethical standards of the relevant national and institutional committees on human experimentation and with the Helsinki Declaration of 1975, as revised in 2008.

## Results

### Participants

In all, 25 FND patients and 24 age- and gender-matched HV completed the study. Participant characteristics are described in Table 1, with FND subjects displaying significantly higher depression and anxiety scores than controls.

**Table 1.** Patient characteristics

	FND^a^		HV				
	*n*	Mean (s.d.)	*n*	Mean (s.d.)	Statistic	df	*p*
Gender, *n*					χ^2^ = 3.20	1	>0.07
Women	22		17				
Men	3		7				
Age, years	25	41.6 (12.2)	24	40.7 (15.5)	*t* = 0.23	47	>0.07
BDI-II	23	22.6 (10.8)	20	6.45 (6.1)	*t* = 6.13	35.6	<0.001
STAI	22	45.4 (14.2)	20	37.5 (9.9)	*t* = 2.08	37.6	0.041
Pain	17	2.6 (0.7)					
Positive motor symptoms	13	2.1 (0.5)					
Negative motor symptoms	20	3.2 (0.8)					
Non-epileptic seizures	10	3.0 (1.0)					
Sensory symptoms	19	2.0 (0.7)					

FND, Functional neurological disorder; HV, healthy volunteers; s.d., standard deviation; df, degrees of freedom; BDI-II, Beck Depression Inventory-II; STAI, Spielberger State-Trait Anxiety Inventory.

^a^ FND symptom severity scores were available in 24 patients and were rated from 1 (mild) to 4 (very severe).

Symptom patterns and severity scores were available for 24 out of 25 FND patients (Table 1). Of the patients, 17 reported pain symptoms (headache *n* = 13, legs *n* = 3, body *n* = 5) with a mean duration of 54.5 (s.d. = 64.2) months. Of the patients, 13 had positive motor symptoms (myoclonic jerks *n* = 2, tremor *n* = 7, dystonia *n* = 3, gait abnormality *n* = 3), and 20 patients reported negative motor symptoms of weakness (lower extremities *n* = 7 or upper and lower extremities, *n* = 13). Average duration of motor symptoms was 53.5 (s.d. = 44.2) months (omission in one subject). Of the patients, 19 stated sensory symptoms (loss of sensation, numbness, pins and needles *n* = 14, tinnitus *n* = 3, hearing loss *n* = 1, double vision *n* = 3, blurred vision *n* = 8, loss of vision *n* = 2) with a mean duration of 46.8 (s.d. = 42.6) months (omission in five subjects). Other symptoms such as stutter (*n* = 4), dysarthria (*n* = 8), dysphonia (*n* = 4), swallowing (*n* = 8), memory (*n* = 10), gastrointestinal (*n* = 6), genitourinary (*n* = 10) and cardiovascular symptoms (*n* = 10) were also reported. Most participants had overlapping symptom profiles. For instance, when considering only positive motor, non-epileptic or weakness symptoms, 16 had mixed symptoms, four had only weakness, two had only non-epileptic seizures and two had only positive symptoms. Of the patients, 12 had a symptom affecting the left upper limb they were using to button press. Eight patients had a symptom affecting the right upper limb that they were using to indicate W or M with a mouse. All subjects were watched carefully to ensure that the symptoms did not interfere with their capacity to perform the experiment. One might also anticipate that the symptom might similarly affect both W and M which were contrasted.

Two participants had current depression of mild severity, 10 additional subjects had a history of depression, two had panic disorder and two had a history of obsessive–compulsive disorder. Medication use included antidepressants (*n* = 17), pregabalin (*n* = 5), gabapentin (*n* = 1), lamotrigine (*n* = 2), topiramate (*n* = 1) and a synthetic opioid (*n* = 1).

### Behavioural results

Average latencies of the W and M judgements relative to the recorded button press were calculated for each individual after excluding outlier trials (> 2 s.d. from the individual mean). There was no significant difference in the number of excluded trials in HV and FND patients (mean 3.7, s.d. = 1.2 *v*. 4.1, s.d. = 1.1, *p* = 0.21). Two outlier FND patients (>3 s.d. from mean across both groups) were excluded from the behavioural data analysis, thus 22 FND patients and 24 HVs were included in behavioural data analysis.

A mixed-design analysis of variance (ANOVA) with the factors judgement (W, M) and group (FND, HV) showed significant main effects of group (*F*_1,45_ = 16.54, *p* < 0.001) and judgement (*F*_1,45_ = 33.79, *p* < 0.001), and a significant interaction (*F*_1,45_ = 4.38, *p* = 0.042). FND patients showed delayed W [FND −99 (s.d. = 125) ms, 95% confidence interval (CI) −163 to −35 ms; HV −252 (s.d. = 175) ms, 95% CI −315 to −190 ms] and M [FND −15 (s.d. = 79) ms, 95% CI −43 to +14 ms; HV −72 (s.d. = 56) ms, 95% CI −100 to −44 ms] judgements compared with HV. The interval between W and M judgements, W–M, was used as an implicit measure of conscious awareness of volitional intention. An independent-samples *t* test demonstrated that FND patients exhibited a significantly reduced W–M interval compared with HV [FND −76 (s.d. = 80) ms; HV −180 (s.d. = 180) ms, *t*_32.3_ = 2.57, *p* = 0.015].

Individual standard deviations across trials were compared which assess the reporting precision of the W judgement. FND subjects reported the W judgement (average s.d. = 156 ms) more precisely than HV (average s.d. = 305 ms, *t*_42.3_ = −2.61, *p* = 0.013), but not the M judgement (FND average s.d. = 135 ms; HV average s.d. = 80 ms, *t*_27.9_ = 1.95, *p* = 0.06).

Exploratory one-way ANOVAs were used to compare the W–M measure of FND patients divided as a function of neurological symptom presentation with HV, showing significant differences for all symptom categories (Table 2). *Post-hoc* analyses showed that FND patients with any kind of motor symptoms had more reduced W–M intervals than HV. To assess the specificity of these findings we compared those with only negative motor symptoms, positive symptoms (with or without negative) and HV. The one-way ANOVA showed a trend (*p* = 0.052); the *post-hoc* analysis showed that patients with positive motor symptoms differed from HV (*p* = 0.019), but not those with only negative symptoms (*p* > 0.2) (Bonferroni correction *p* < 0.025). Note that all subjects with positive symptoms also had negative symptoms. We attempted to examine laterality for positive motor symptoms but only three subjects had positive symptoms that did not affect the hand used in the task. We compared subjects with bilateral/left-sided weakness [*n* = 8, −49 (s.d. = 52) ms] and right-sided weakness [*n* = 8, −82 (s.d. = 75) ms] with HV [*n* = 20, −178 (s.d. = 174) ms], which showed a trend using a one-way ANOVA (*p*=0.054).

**Table 2.** W and M judgements as a function of functional symptom type^a^

	Present		Absent		HV			
Symptom	*n*	Mean (s.d.)	*n*	Mean (s.d.)	Mean (s.d.)	*F* (*p*)	Present *v*. HV: *p*	Absent *v*. HV: *p*
Positive motor	12	−55 (78)	10	−87 (66)	−180 (180)	3.58 (0.037)	0.017	0.100
Negative motor	18	−79 (78)	4	−26 (25)	−180 (180)	3.69 (0.033)	0.029	0.051
Positive motor *v*. only negative motor	12	Positive (with/without negative) −55 (78)	8	Negative only −101 (67)	−180 (180)	3.18 (0.052)	Positive (with/without negative) 0.019	Negative only 0.211
Non-epileptic seizures	8	−49 (64)	14	−79 (78)	−180 (180)	3.55 (0.037)	0.037	0.040
Somatosensory	13	−81 (63)	9	−53 (85)	−180 (180)	3.55 (0.038)	0.054	0.027

Data are given as mean difference between W and M judgements (in ms) (s.d.).

HV, Healthy volunteers; s.d., standard deviation; ANOVA, analysis of variance; df, degrees of freedom.

^a^ *F* values of the ANOVAs are given, as well as *p* values of the ANOVAs and Fisher's least significant difference *post-hoc* tests. All ANOVAs had df_factor_ = 2 and df_error_ = 43, except the ANOVA comparing functional neurological disorder patients with positive motor symptoms, patients with only negative symptoms and HV, which had df_factor_ = 2 and df_error_ = 41.

### fMRI results

We excluded two FND patients and two HV from the analysis due to excessive head movement (>0.5 mm), where over one-third of volumes needed repairing with ArtRepair (Mazaika *et al.* 2007). Data from 23 FND patients and 22 HV were included in the fMRI analysis; in the comparison between groups, data were also analysed after excluding the two outlier patients identified in the behavioural analysis, and all fMRI findings were consistently observed.

**Fig. 2.**
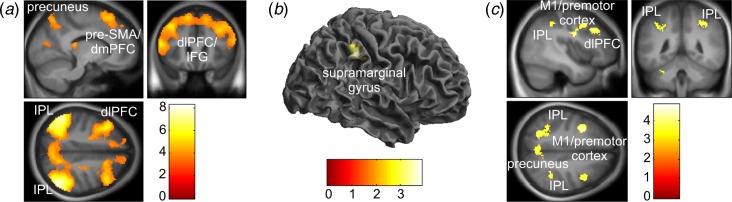
Attention to intention *v*. movement. (*a*) Significant activations associated with attention to intention compared with attention to movement (intention *v*. movement contrast) across all participants (*n* = 45). (*b*) Regions of significantly decreased activity for functional neurological disorder patients (*n* = 23) compared with healthy volunteers (*n* = 22) when attending to intention compared with attending to movement. Image displayed at *p* < 0.005 (uncorrected) for illustration. (*c*) Results of the correlation between the intention *v*. movement contrast and the behavioural measure W-M across all participants (*n* = 45). Image displayed at *p* < 0.001 (uncorrected) for illustration. pre-SMA, Pre-supplementary motor area; dmPFC, dorsomedial prefrontal cortex; IPL, inferior parietal lobule; dlPFC, dorsolateral prefrontal cortex; IFG, inferior frontal gyrus; M1, primary motor cortex.

Across all participants, attention to intention relative to attention to movement was associated with increased activity in the bilateral IPL, dlPFC/inferior frontal gyrus and pre-SMA/dlPFC, as well as some minor clusters within temporal and occipital lobes (Fig. 2*a*, online Supplementary Table S1).

When comparing intention *v*. movement trials between groups, FND patients exhibited significantly reduced activity within the right IPL [supramarginal gyrus, Brodmann area (BA) 40; peak voxel reported in MNI coordinates *x y z* in mm: 54, −32, 42; cluster size = 36; *Z* = 3.58] compared with HV (Fig. 2*b*). The above-mentioned results were replicated after excluding the two outlier patients: FND patients showed significantly reduced IPL activity with the same peak coordinates (cluster size = 43; *Z* = 3.70) compared with HV. An additional activation in the left premotor cortex was also revealed (superior frontal gyrus; MNI coordinates: −18, 6, 50; cluster size = 49; *Z* = 3.67).

The W–M interval was positively correlated with BOLD activity in intention *v*. movement trials in the bilateral IPL and primary motor/premotor areas, and left dlPFC and precuneus (Fig. 2*c*, online Supplementary Table S2).

### rsfMRI results

**Fig. 3.**
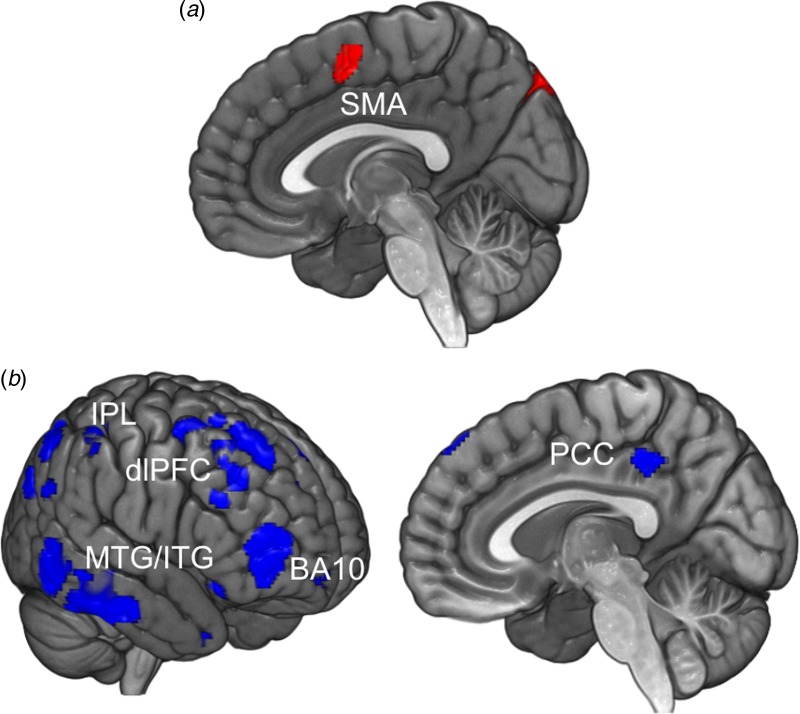
Resting-state functional connectivity from right inferior parietal cortex (IPC) seed. (*a*) Increased (functional neurological disorder patients > healthy volunteers; FND>HV) and (*b*) decreased (HV > FND) functional connectivity from IPC to whole brain for FND patients (*n* = 25) compared with HV (*n* = 70) during rest. Image displayed at *p* < 0.005 (uncorrected) for illustration. SMA, Supplementary motor area; IPL, inferior parietal lobule; dlPFC, dorsolateral prefrontal cortex; MTG, middle temporal gyrus; ITG, inferior temporal gyrus; BA, Brodmann area; PCC, posterior cingulate cortex.

Data from 25 FND patients and 70 HV [40 women, 40.19 (s.d. = 12.70) years old], including those HV that took part in the fMRI study, were included in the rsfMRI analysis. Compared with HV, FND patients demonstrated reduced connectivity between the right IPC and frontal control regions [dlPFC, anterior cingulate cortex (ACC), BA 10; Fig. 3*b*], but increased functional connectivity with the premotor cortex and SMA (Fig. 3*a*, online Supplementary Table S3).

## Discussion

We show using Libet's clock task (Libet *et al.* 1983) that FND is characterized by impaired awareness of voluntary motor intention with delayed W relative to M judgements. The judgement of W was both abnormally delayed and more precise relative to HV. On an exploratory basis, this delay in W was particularly evident in patients with involuntary functional positive motor symptoms and less so in those with only negative motor symptoms. Our results concur with a previous report of abnormalities in the conscious experience of action in a small sample of 11 patients with psychogenic tremor (Edwards *et al.* 2011), replicating these findings in a larger sample. These findings might also explain observations of impaired effortful voluntary movement in FND (Criswell *et al.* 2010) and common observations of enhanced attention towards both voluntary and involuntary movement. As expected, across all subjects, the attention to intention *v*. movement was associated with enhanced activity in the bilateral IPL, dlPFC and pre-SMA. Crucially, FND subjects had lower activity in the right IPL (BA 40, supramarginal gyrus) compared with HV in the contrast of W–M. This region was also positively correlated with the behavioural measure W–M across all subjects, thus emphasizing its role in the conscious awareness of volitional intention. The network implicated in voluntary motor intention also showed aberrant functional connectivity at rest in FND subjects. FND showed reduced resting-state functional connectivity between the right IPC and prefrontal structures (dlPFC, ACC, BA 10), regions upstream in the processing of intention and action selection, but increased connectivity with the premotor cortex and SMA, regions downstream in the processing of intention and motor preparation. Previous studies in FND have implicated lower TPJ/IPC activity to involuntary functional symptoms (Voon *et al.* 2010) and lower SMA activity during voluntary motor preparation (Voon *et al.* 2011). Here we specifically isolate the awareness of voluntary intention and build on data implicating an abnormal fronto-parietal network and suggest that these findings might play a role in the subjective sense of involuntariness.

Our findings may implicate a general impairment in inferior parietal function. Previous studies have shown hypoactivity of the right TPJ in the comparison of involuntary and mimicked tremor (Voon *et al.* 2010). As neural regions associated with the sensory outcome appeared to be intact, we had speculated that the primary deficit might be related to an impairment at the level of the sensory prediction (Voon *et al.* 2010). A follow-up study further showed decreased functional connectivity of the right TPJ seed using average coordinates from a meta-analysis of self-agency studies (peak *x y z* = 51, −46, 31 mm) within BA 39 (Maurer *et al.* 2016). The TPJ is a large region encompassing the IPL. The right IPL activation in the attention to intention *v*. movement condition across all subjects was very large (cluster size *k* = 10 439) and included the right TPJ. However, the right IPL activation (peak *x y z* = 54, –32, 42 mm) in the group comparison (FND < HV; Figs 2 and 3) is localized in the angular/supramarginal gyrus in BA 40 and distant from the superior temporal lobule. Both BA 39 and BA 40 are implicated in impairments in intention using Libet's clock (Desmurget & Sirigu, 2012). A generalized impairment in inferior parietal cortical function may be an issue which has been observed in some but not all FND studies (for a review, see Voon *et al.* 2016). Differences in imaging task design and hence function might thus highlight different parietal regions. Thus, although both studies implicate impairments in the IPC, the focus on intention in voluntary movements implicates BA 40 whereas differences between the comparison of involuntary and voluntary movement might highlight more comparator systems of sensory prediction and outcome implicating BA 39 or the TPJ.

### Mechanistic considerations

There are several possible explanations for our findings. Here we discuss plausible mechanisms including the role of attention, impairments in explicit intentional processes, decision thresholds and movement veto.

Attending to and reporting the intention to move might be more difficult and demanding more attention than reporting the movement itself, so that subjects with attentional impairments may have more difficulty in making the W judgement. Non-specific impairments in attention have been documented across some but not all studies in FND (for a review, see Voon *et al.* 2016). However, in our study, FND patients reported the W judgement more precisely than HV, thus suggesting that the reduced latency is unlikely to be due to a non-specific attentional impairment. We also controlled for non-specific attention effects by comparing two conditions that require the allocation of attention to either intention or movement. An intriguing follow-up of this study might be to consider how W–M might present in subjects with malingering in which the symptoms are deliberately produced. One might speculate that the variability associated with reporting of W might be much greater than observed in FND.

Theoretical models and empirical evidence suggest enhanced directed attention towards self-related representations and pathological symptoms (Brown, 2004; Cojan *et al.* 2009; Edwards *et al.* 2012). These findings of an enhanced precision (inverse variance) of abnormally delayed voluntary intention converge with theories of enhanced attention towards and thus precision of abnormal pathological priors or expectations in FND (for a review, see Edwards *et al.* 2012). Although enhanced attention to the movement symptom is observed in FND, what is not clear is whether this is a primary pathology or possibly a compensatory mechanism underlying primary impairments in volitional processes. Enhanced attention to both voluntary and functional movements is observed clinically in FND (van Poppelen *et al.* 2011) and attention appears to play a role in the expression of the symptom as demonstrated by clinical signs of improvement or cessation of functional symptoms with distractability or shifts of attention away from the functional movement (Schwingenschuh & Deuschl, 2016). Attention focused on a novel voluntary movement is required for optimal motor function but can impair automatic overlearned habitual movements. That attention is enhanced to self in FND might be compensatory and secondarily related to primary impairments in voluntary motor intention. The enhanced attention might then also interfere with skilled automatic movements. That focused attention increases the functional movement suggests that the aberrant movement is novel and goal-oriented in nature rather than automatic and habitual as otherwise, attention should impair habits and decrease the functional movement. Our findings would be consistent with clinical observations of increased attention towards both the involuntary and voluntary movements in FND.

Several lines of evidence suggest impairments in explicit but not implicit motor processes in FND, suggesting possible impairments in motor conceptualization or volition, first theorized by Spence *et al.* (2000) (for a review, see Voon *et al.* 2016). FND subjects are impaired at explicit instructed *v*. implicit automatic mental rotation of hands or feet (Roelofs *et al.* 2001). In this study, functional paralysis subjects subjectively reported that they could not explicitly mentally rotate their hands or feet to match the image in 51% of foot trials, compared with 0% in controls. Similarly, FND subjects were impaired in tasks dissociating explicit pre-planned movements under full control or under greater certainty but intact in implicit movements occurring more automatically under contexts in which subjects may be less aware (e.g. one-back reaching, visuomotor transformation or variable predictive value of precued reaction time) (Parees *et al.* 2013). Focusing on the motor intention phase of movement, FND subjects also show decreased activity in the SMA and increased activity in limbic regions (amygdala, anterior insula) relative to healthy controls (Voon *et al.* 2011). FND was also associated with decreased functional connectivity between the dlPFC and SMA in freely chosen *v*. directed cued movement, suggesting a possible impairment in higher-order action selection during freely chosen actions. Together these findings suggest primary impairments in explicit motor intentional processes with intact implicit automatic processes. These findings help explain observations of impaired voluntary movement in FND as tested on finger tapping (Criswell *et al.* 2010) and common clinical observations of enhanced attention and effort directed towards voluntary movement which may be secondary to the primary impairment. Indeed, a supportive clinical criterion of functional movement is slowed effortful voluntary movement (Schwingenschuh & Deuschl, 2016). The mechanisms underlying functional movement may also represent an extension of this impairment in explicit volitional motor process engaging enhanced attention. The enhanced attention may also then secondarily interfere with skilled automatic movements.

These abnormalities in intention awareness appear to be greater in patients with positive motor symptoms, although we note that the relationship with specific neurological symptoms should be interpreted cautiously given the limited sample size. Nevertheless, several intriguing mechanisms may be specifically relevant to positive motor symptoms. It has been suggested that movements are produced when a certain decision threshold is crossed and that the precise moment at which this threshold is crossed is largely determined by spontaneous subthreshold fluctuations of neuronal activity (Schurger *et al.* 2012). In the context of FND, it could be speculated that patients might have a lower decision threshold and/or increased neuronal subthreshold fluctuations, resulting in an increased likelihood of abnormally crossing said threshold and thus producing aberrant movements. Indeed, the *Bereitschaftspotential*, which has been posited to reflect the increase in spontaneous neuronal subthreshold fluctuations preceding voluntary movements (Schurger *et al.* 2012), has been found to precede psychogenic myoclonus (Terada *et al.* 1995).

Another possible underlying mechanism could be related to the interval between awareness of intention and movement, which has been hypothesized to temporally allow for a conscious inhibition or veto of the movement (Libet, 1999; Haggard & Libet, 2001), as well as for an evaluation of whether the selected action might be optimal to obtain the desired effect (Haggard & Libet, 2001). A recent study (Schultze-Kraft *et al.* 2016) reported that initiated movements could be inhibited if a stop signal occurred up to 200 ms prior to movement onset, but not if it occurred under that time. Since the temporal shift of intention awareness towards the movement in FND patients is well under 200 ms, it could suggest that this results in a decrease in the time available and likelihood of action inhibition or movement veto. Still, this remains speculative based solely on the data at hand. However, as in the current study, shortened W–M intervals have been mainly observed in neurological and neuropsychiatric disorders characterized by positive symptomatology (Edwards *et al.* 2011; Moretto *et al.* 2011; Tabu *et al.* 2015). These findings converge with a study of FND focusing on positive motor symptoms in which the only cognitive deficit observed across a range of tasks was impaired response inhibition on a Go/NoGo task (Voon *et al.* 2013). Alternatively, the findings may also be related to prior experience with functional movements, which may secondarily result in impaired reliance on the subjective awareness of the intention to move.

### IPC

Our findings implicate the IPC, but, contrary to our hypothesis, not the SMC. Cortical stimulation of the IPC (BA 39/40) has been associated with subjective feelings of ‘wanting to move’ a body part, whereas stimulation of the SMC results in an uncontrollable ‘urge’ to produce a specific movement (Desmurget *et al.* 2009; Desmurget & Sirigu, 2012). This phenomenological difference suggests the intentional feelings evoked in the IPC may lie upstream of the SMC, and has been hypothesized to reflect the role of the IPC in pure intention (Desmurget *et al.* 2009). However, which of these structures is key to voluntary action remains controversial. Some authors attribute this role to the pre-SMA, highlighting its involvement in transforming thoughts into actions (Haggard, 2008), whereas others emphasize evidence suggesting that the locus of consciousness might be within posterior brain regions (Koch *et al.* 2016). Damage to both fronto-mesial and IPC regions has been associated with the alien hand syndrome, in which movements are expressed outside of volitional control (Scepkowski & Cronin-Golomb, 2003). In an fMRI study of a patient with a posterior parietal lesion, alien hand movement was associated with circumscribed activity of the contralateral primary motor cortex, which was suggested to arise in the absence of volitional awareness from the IPC (Assal *et al.* 2007). Indeed, in patients with IPC stroke lesions, the latency of the W judgement is shifted towards movement onset as compared with HV and cerebellar patients (Sirigu *et al.* 2004). Together these findings suggest a role for the IPC at an early stage of motor intention.

Another possible parallel lies in studies of motor imagery or internal rehearsal of motor representations. Recently, a tetraplegic patient was shown to be able to control posterior parietal activity though motor imagery (Aflalo *et al.* 2015). Recordings showed that neurons in this area code both goal and imagined trajectory of the movements, indicating that this region encodes motor intention and that its signals could be used for neuroprosthetic applications (Aflalo *et al.* 2015). Motor imagery implicates similar neural regions as action execution (Decety *et al.* 1994; Sirigu *et al.* 1996) and has been studied in FND as discussed above. In a limb rotation task (Roelofs *et al.* 2001), participants with functional paralysis showed no difference to HV when judging whether images of rotated hands and feet corresponded to the right or left limb. However, they were slower than HV when explicitly asked to mentally rotate their own limbs to match the position of the image, with patients subjectively reporting impaired motor imagery in that they had difficulty mentally rotating their limbs to match the image whereas no difficulties were reported in controls. These impairments in explicit motor imagery prior to and without actual movement led the authors to emphasize an impairment in intentional processes in FND (Roelofs *et al.* 2001).

### Limitations and conclusion

The study is not without limitations. Although we specifically recruited subjects that were able to remain still in the MRI environment, it cannot be completely ruled out that the subjects’ neurological symptoms interfered with testing and that weakness or functional movements occurred during scanning. However, as these symptoms may have occurred randomly across the blocks, the contrast used should effectively cancel any neural activity from adventitious symptoms. Larger sample sizes with greater symptom specificity would also be helpful in understanding the relationship of this measure with specific symptoms. Furthermore, how these findings might be influenced by other symptoms commonly observed in FND including panic symptoms or other somatoform disorders remains to be established. The majority of our sample was also on medications, which is commonly observed in FND cohorts. The use of medications might interfere with imaging findings; further studies in other samples on such chronic medications might be indicated. Co-morbid symptoms of depression or anxiety are also very common in FND, and the effect of depression or anxiety symptoms cannot be ruled out. When BDI and STAI are added as covariates in ANOVA in behaviour analysis, significant interactions between conditions (intention or movement) *v*. BDI or STAI were observed and the main effect of groups or conditions was no longer observed. FND is also commonly associated with generalized anxiety which was not assessed in this study.

Despite the main findings of Libet *et al.* (1983) having been widely replicated (Lau *et al.* 2004; Edwards *et al.* 2011; Fried *et al.* 2011), including in the current study, Libet's clock task has not been exempt of criticism. The precision and nature of the W judgement has been called into question, with authors suggesting that the high attentive demands of the task might result in an imprecise measurement of the internal process (Stamm, 1985), or that the W judgement might represent a peak of intention (Ringo, 1985), meaning that the subject's intention would in reality precede the reported urge, in contrast to the simultaneity posited by Libet *et al.* (1983). Recent studies suggest that the decision for a movement does not abruptly appear in a binary manner but is gradually constructed; thus Libet's task may involve a cut-off for binary translation of the continuous preparation of action decision (Guggisberg & Mottaz, 2013). In addition, that subjective time of intention onset is influenced by perceptual information such as sensory feedback (Guggisberg & Mottaz, 2013; Wolpe & Rowe, 2014). Introspection to one's own intention might be also intermittent or rather retrospectively inferred. Critics thus implied that the unconscious nature of the movement intention, as reflected by the *Bereitschaftspotential* preceding intention awareness, would not be such (Stamm, 1985). Haggard & Libet (2001), however, countered that even the largest estimates of the prior entry effect, by which attention affects the judgement of synchrony of events occurring in different streams, are an order of magnitude smaller than the 200 ms interval between *Bereitschaftspotential* and W judgement posited in the original work (Libet *et al.* 1983). Moreover, even those who questioned the precision or validity of the absolute W and/or M judgements conceded that their criticism might not affect the relative distance between them (Rollman, 1985). The latter is of particular importance to the current work, as it is this interval that is postulated to be used by the subject to monitor the desirability and effect of the action (Libet's veto) and whether it is an optimal action plan (Haggard's specificity) (Haggard & Libet, 2001). Therefore, irrespective of the conscious nature of the movement, a reduced veto period would have a higher likelihood of resulting in maladaptive actions, such as the aberrant behaviours observed in our FND patients and reported by previous researchers (Edwards *et al.* 2011; Moretto *et al.* 2011; Tabu *et al.* 2015).

In conclusion, our findings build on reports suggesting impairments in motor intention awareness in FND and specifically highlight abnormalities of the early motor processing network focusing on the right IPL. These findings might explain common clinical observations of enhanced attention towards movements in FND and impaired effortful voluntary movements and provide possible novel targets for therapeutic intervention that might include psychological or physiological interventions or neuromodulation.

## Supplementary material

For supplementary material accompanying this paper visit http://dx.doi.org/10.1017/S0033291717000071. click here to view supplementary material
