# Supplementary material for: Impaired awareness of motor intention in functional neurological disorder: implications for voluntary and functional movement
Source: Psychol Med. 2017 Feb 10;47(9):1624–36. doi: 10.1017/S0033291717000071 (PMC5964459; doi:10.1017/S0033291717000071)
Supplement: Supplementary file 1 [file S0033291717000071sup001.doc]

**Supplementary material**

**Supplementary Table S1.** Brain regions associated with attention to intention

Significant BOLD activation associated with attention to intention as compared to attention to movement for all participants (*n*=45). Cluster-extent threshold-correction, 15 voxels, *p*<.001 (uncorrected). BA: Brodmann area; dlPFC: dorsolateral prefrontal cortex; IPL: inferior parietal lobule; *k*: cluster extent.

| Peak region | x | y | z | *k* | *Z* |
| --- | --- | --- | --- | --- | --- |
| R IPL (BA 40) | 52 | -54 | 48 | 10,439 | 6.41 |
| L IPL | -52 | -52 | 48 |  | 6.37 |
| L dlPFC/inferior frontal gyrus | -56 | 22 | 14 | 10,322 | 5.16 |
| R cuneus (BA 17) | 9 | -90 | 2 | 1,171 | 4.58 |
| L middle temporal gyrus | -64 | -48 | 4 | 653 | 4.39 |
| R superior temporal gyrus (BA 22) | 68 | -38 | 4 | 259 | 4.37 |
| R middle temporal gyrus (BA 21) | 44 | 8 | -34 | 290 | 4.19 |
| R inferior frontal gyrus | 48 | 36 | -8 | 839 | 4.04 |
| L middle temporal gyrus | -54 | -2 | -18 | 167 | 4.02 |
| L middle occipital gyrus | -20 | -92 | 4 | 122 | 4.02 |
| L calcarine sulcus | -18 | -74 | 12 | 106 | 3.67 |

**Supplementary Table S2.** Brain regions correlated with the length of the W-M interval

Regions showing significant correlations between the Intention vs. Movement contrast and the behavioural measure W-M across all subjects (*n*=45). Cluster-extent threshold-correction, 15 voxels, *p*<.001 (uncorrected). BA: Brodmann area; dlPFC: dorsolateral prefrontal cortex; IPL: inferior parietal lobule; *k*: cluster extent.

| Peak region | *x* | *y* | *z* | *k* | *Z* |
| --- | --- | --- | --- | --- | --- |
| L premotor cortex/M1 | -34 | 4 | 38 | 204 | 4.31 |
| R premotor cortex/M1 | 42 | -8 | 28 | 228 | 4.25 |
| L precuneus (BA 7) | -4 | -62 | 48 | 541 | 4.04 |
| L cerebellum (dentate nucleus) | -20 | -60 | -32 | 157 | 4.00 |
| R IPL | 36 | -44 | 44 | 111 | 3.99 |
| R calcarine gyrus (BA 31) | 18 | -66 | 12 | 318 | 3.90 |
| L superior parietal lobule | -28 | -62 | 44 | 285 | 3.82 |
| R dlPFC/middle frontal gyrus | 42 | 26 | 36 | 222 | 3.75 |
| R premotor cortex/superior frontal gyrus | 20 | 2 | 46 | 68 | 3.68 |
| L superior occipital gyrus (BA 18) | -14 | -90 | 12 | 87 | 3.63 |
| L premotor cortex/superior frontal gyrus | -20 | 0 | 50 | 39 | 3.63 |
| L superior frontal gyrus | -16 | 36 | 32 | 41 | 3.59 |
| L dlPFC (BA 9) | -42 | 24 | 34 | 35 | 3.49 |

**Supplementary Table S3.** Results of the resting-state functional connectivity analysis

Resting-state functional connectivity of FND patients compared to HV. Cluster-extent threshold-correction, 15 voxels, *p*<.001 (uncorrected). BA: Brodmann area; dlPFC: dorsolateral prefrontal cortex; IPL: inferior parietal lobule; *k*: cluster extent; SMA: supplementary motor area.

| Peak region | x | y | z | *k* | *Z* |
| --- | --- | --- | --- | --- | --- |
| FND>HV |  |  |  |  |  |
| R cuneus | 15 | -86 | 44 | 229 | 4.75 |
| R cerebellum (VIII) | 31 | -65 | -54 | 32 | 4.2 |
| L cerebellum (VI) | -13 | -69 | -21 | 32 | 3.95 |
| L cuneus (BA 19) | -15 | -86 | 42 | 80 | 3.95 |
| R SMA | 6 | 3 | 56 | 27 | 3.82 |
| L middle occipital gyrus (BA 19) | 43 | -88 | 2 | 16 | 3.74 |
| R premotor cortex (BA 6) | 55 | 0 | 35 | 18 | 3.44 |
| FND<HV |  |  |  |  |  |
| R middle temporal gyrus | 62 | -25 | -19 | 448 | 4.78 |
| R inferior temporal gyrus (BA 37) | 64 | -55 | -14 | 117 | 4.55 |
| R dlPFC/ middle frontal gyrus | 31 | 28 | 35 | 399 | 4.53 |
| R IPL | 41 | -65 | 58 | 122 | 4.40 |
| R middle frontal gyrus | 38 | 52 | 2 | 185 | 4.37 |
| R IPL (BA 39) | 43 | -69 | 30 | 96 | 4.09 |
| R premotor cortex (BA 6) | 36 | 7 | 60 | 51 | 3.71 |
| L cerebellum (crus I) | -48 | -74 | -33 | 17 | 3.64 |
| R IPL (BA 39) | 48 | -55 | 32 | 30 | 3.61 |
| R medial frontal gyrus | 20 | 45 | -3 | 16 | 3.53 |
